# Trait-mediated linkages and stocking rate thresholds in plant-community-soil feedbacks to long-term grazing in a *Stipa breviflora* desert steppe

**DOI:** 10.3389/fpls.2026.1757984

**Published:** 2026-03-02

**Authors:** Jiangwen Li, Yiwei Feng, Liping Li, Xiaoxi Zhang, Meiyan Fang, Qirui Ye, Jiahui Hao, Guodong Han

**Affiliations:** 1 College of Life Sciences, Yan’an University, Yan’an, China; 2 Key Laboratory of Grassland Management and Utilization of Inner Mongolia, Inner Mongolia Agricultural University, Hohhot, China; 3 Key Laboratory of Forage Cultivation, Processing and High Efficient Utilization of the Ministry of Agriculture and Rural Affairs, Inner Mongolia Agricultural University, Hohhot, China; 4 Key Laboratory of Grassland Resources of the Ministry of Education, College of Grass-land Science, Inner Mongolia Agricultural University, Hohhot, China

**Keywords:** desert steppe, functional traits, plant community, soil nitrogen, *Stipa breviflora*, stocking rate

## Abstract

**Introduction:**

*Stipa breviflora* desert steppe serves as a critical ecological barrier in arid and semi-arid regions, where grazing represents the dominant land use practice. The regulation of stocking rate directly determines ecosystem structural stability and functional integrity. However, existing research has primarily focused on single components or short-term responses, lacking systematic analysis of multi-component synergistic responses under long-term gradient stocking rates.

**Methods:**

This study utilized the Inner Mongolia *S. breviflora* desert steppe long-term grazing control experimental platform initiated in 2004, establishing four stocking rate treatments. We systematically measured community quantitative characteristics, multi-organ functional traits of *S. breviflora*, and soil physicochemical indicators. Data were analyzed using one-way ANOVA, Pearson correlation analysis, principal component analysis, and linear/nonlinear regression models.

**Results:**

Results demonstrated that species richness exhibited a unimodal (quadratic) response to stocking rate, with the highest richness under LG, followed by CK, and significant declines under MG and HG. Community aboveground biomass and height decreased consistently with increasing stocking rate. Concurrently, *S. breviflora* enhanced its relative dominance via coordinated multi-organ trait adjustments, characterized by reduced aboveground stature and leaf biomass, coupled with increased root branching and volume. Analysis of the multi-component system revealed nonlinear relationships among community, plant, and soil variables. Principal component analysis revealed a clear separation between ungrazed and grazed treatments along the primary axis, whereas MG and HG treatments converged, indicating a stocking rate threshold beyond which ecosystem responses became saturated. Soil ammonium nitrogen demonstrated significant positive effects on species richness, whereas soil nitrate nitrogen and bulk density exhibited significant inhibitory effects.

**Discussion:**

This study advances the theoretical framework of multi-component coupling in arid grassland ecosystems, providing direct scientific support for determining critical stocking rates and implementing precise restoration of degraded grasslands in Inner Mongolia *S. breviflora* desert steppe.

## 1 Introduction

Desert steppe ecosystems constitute critical ecological security barriers in arid and semi-arid regions, while simultaneously serving as fundamental carriers for sustainable livestock husbandry. Their structural stability and functional integrity directly influence regional ecological equilibrium and global climate change responses (Li et al., 2024). *Stipa breviflora* desert steppe represents the dominant community type within Inner Mongolia’s desert grasslands, providing essential ecosystem services including wind erosion control, carbon sequestration, and high-quality forage production. Grazing, as the predominant land use practice in these regions, relies on appropriate stocking rate regulation as the core mechanism for maintaining grassland ecosystem equilibrium (Lv et al., 2021). Research has demonstrated that grazing disturbance directly influences structural and functional evolution of grassland ecosystems by altering plant community competition patterns and resource allocation strategies (Zhang et al., 2018). Furthermore, tight coupling relationships exist among community structure, plant functional traits, and soil physicochemical processes within desert steppe ecosystems, where changes in any single component may trigger system-wide cascade effects—a principle that has become established consensus within grassland ecology research (Chai et al., 2019).

Although extensive research has been conducted on grazing effects on grassland ecosystems, significant knowledge gaps remain. Previous studies have demonstrated that light grazing can enhance community species diversity by alleviating interspecific competition pressure from dominant species, whereas heavy grazing leads to community simplification through competitive exclusion (Lv et al., 2024). Research on *S. breviflora* has primarily focused on aboveground trait responses, suggesting that this species reduces herbivory risk and maintains survival through phenotypic adjustments such as reduced stature and increased tiller production (Li et al., 2022). Soil-focused studies indicate that livestock trampling under grazing disturbance increases soil bulk density and alters nutrient mineralization rates, thereby constraining plant resource acquisition efficiency (Valani et al., 2025). However, existing research has predominantly examined single-component or single-scale responses, lacking systematic analysis of multi-component synergistic responses under long-term gradient stocking rates (Liu et al., 2011; Wang et al., 2018). Current understanding remains limited regarding how dominant species *S. breviflora* adapts to different grazing pressures through coordinated phenotypic plasticity among aboveground morphology, leaf stoichiometry, and belowground root traits. Furthermore, the intrinsic linkage mechanisms among community characteristics, plant multi-organ traits, and soil nutrient cycling, as well as how stocking rate threshold effects regulate these relationships, remain insufficiently understood. This limitation hinders a comprehensive understanding of the mechanisms driving desert steppe ecosystem evolution and stability maintenance under long-term grazing disturbance (Ju et al., 2024). Therefore, this study aims to address the following scientific questions: (1) What are the specific linkage relationships among community species diversity, multi-organ traits of *S. breviflora*, and soil nutrient cycling under long-term gradient stocking rates? (2) How do stocking rate thresholds regulate the response direction and intensity of these linkage relationships? (3) Is the coordinated multi-organ trait response of *S. breviflora* the key functional nexus connecting changes in community structure and soil process?

To address these questions, this study employed the long-term grazing control experiment established in 2004 in Inner Mongolia’s *S. breviflora* desert steppe as the research platform. All measurements were conducted during a single growing season (August 2024). However, as this sampling followed over two decades of consistently applied grazing treatments, the observed patterns are interpreted as reflecting the stabilized ecosystem state under long-term grazing equilibrium, rather than transient responses to interannual environmental variability. We implemented four different stocking rate gradients and systematically quantified community quantitative characteristics, individual functional traits of the dominant species *S. breviflora*, and physicochemical indicators of grazing pasture soils. The core innovation lies in systematically revealing the synergistic response patterns of desert steppe under long-term stocking rate gradients from a multi-component coupling perspective of community, plant individual, and soil, thereby overcoming the limitations of single-component studies. Simultaneously, we clarified the regulatory effects of stocking rate threshold effects on ecosystem multi-component linkage characteristics and elucidated the multi-organ trait coordination strategies of *S. breviflora* in adapting to grazing disturbance. In the present study, ecosystem structural stability was characterized by four key indicators: community species richness, density, coverage, and height. Ecosystem functional integrity was quantified using aboveground biomass, relative importance of the dominant species, multi-organ functional traits of plants, and soil nitrogen availability. This set of evaluation indicators provides a clear and operable basis for quantifying and analyzing the structural stability and functional integrity of the *S. breviflora* desert steppe ecosystem.

## 2 Materials and methods

### 2.1 Experimental design

**Figure 1:**
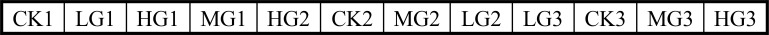
Experimental design of long-term grazing plots in *S. breviflora* desert steppe.

The grazing experiment was initiated in 2004 in an area with uniform topography, vegetation, and soil types. A long-term controlled grazing experiment was established using a randomized complete block design, comprising 3 blocks with 4 grazing treatments each, totaling 12 plots (**Figure 1**). Each grazing plot measured 4.4 hectares. The four treatments consisted of: 0 (control, CK), 0.93 (light grazing, LG), 1.82 (moderate grazing, MG), and 2.71 (heavy grazing, HG) sheep units/hectare/half-year.

Stocking rate standards were established based on field surveys and referenced from research by Wang Mingjiu and Ma Changsheng (Wang and Ma, 1994) on Inner Mongolia desert steppe stocking rates, as well as the theoretical carrying capacity for Inner Mongolia *S. breviflora* grasslands proposed by Wei Zhijun et al (Wei et al., 2000). Grazing was implemented from June 1 to November 30 each year, with no livestock present during the remaining months. Grazing animals consisted of 2-year-old castrated male sheep, replaced every three years. Grazing schedule: animals were released for grazing at 7:00 AM and returned to shelter at 8:00 PM.

Data were collected in August 2024. This single-season snapshot was taken after 20 consecutive years of maintained grazing treatments. Within the context of this long-term experiment, we posit that the measured variables primarily reflect the cumulative and relatively stabilized effects of the different stocking rates, minimizing the confounding influence of short-term climatic fluctuations. Due to the limitation of single-year data collection, this study focuses on the steady-state patterns of species diversity after long-term grazing, while the temporal dynamics will be explored through continuous monitoring in the future.

Additionally, since 2004, ten 1.5m×1.5m exclosure cages were randomly placed within each grazing plot to prevent livestock grazing. Cage positions were randomly relocated annually. No exclosure cages were established in the control plots.

### 2.2 Data collection

Data collection was conducted in August after 20 years of grazing implementation, coinciding with the peak biomass period of plant communities. Within each grazing plot, inside which 1m×1m quadrats were set up to monitor plant community characteristics, including plant height, coverage, density, and biomass. Simultaneously, plant functional traits of the dominant species *S. breviflora* were collected, along with relevant soil physicochemical indicators, as detailed in **Table 1**.

**Table 1:** Terminology and units for community, plant, and soil indicators in *S. breviflora* grassland.

Category	Functional traits	Abbreviation	Unit
Plant Community	Community Coverage	CC	%
	Community Height	CH	cm
	Community Aboveground Biomass	C-AGB	g/m^2^
	Community Density	CD	ind./m^2^
	Species Richness	SR	species/m^2^
*S. breviflora* Structural Traits	Individual Aboveground Biomass of *S. breviflora*	I-AGB	g/m^2^
	Height	H	cm
	Coverage	C	%
	Density	D	ind./m^2^
	Width Of Clump Crown	WCC	cm
	Diameter Of Stem	DS	cm
	Plant Fresh Weight	PFW	g
*S. breviflora* Leaf Traits	Leaf Fresh Weight	LFW	g
	Leaf Dry Weight	LDW	g
	Leaf Area	LA	cm^2^
	Specific Leaf Area	SLA	cm^2^/g
	Plant Leaf Area	PLA	cm^2^
	Individual Leaf Mass	LWE	g
	Leaf Number	LN	NO.
	Leaf Nitrogen Content	LNC	%
	Leaf Carbon Content	LCC	%
	Leaf Phosphorus Content	LPC	%
*S. breviflora* Stem Traits	Stem Fresh Weight	SFW	g
	Stem Dry Weight	SDW	g
	Stem Nitrogen Content	SNC	%
	Stem Carbon Content	SCC	%
	Stem Phosphorus Content	SPC	%
*S. breviflora* Root Traits	Root Length	RL	cm
	Root projected Area	RPA	cm^2^
	Root Surface Area	RSA	cm^2^
	Root Average Diam	RAD	mm
	Root Volume	RV	cm³
	Root Tips Number	RTN	tips/tiller
	Root Furcation Number	RFN	Furcation s/tiller
	Root Crossing Number	RCN	Crossing s/tiller
Soil Surface Physicochemical Properties	Soil Ammonium Nitrogen	NH_4_^+^-N	mg/kg
	Soil Nitrate Nitrogen	NO_3_^−^-N	mg/kg
	Soil Bulk Density	BD	g/cm^2^
	Soil Gravimetric Water Content	θg	%
	Soil Potential of Hydrogen	pH	
	Soil Total Nitrogen	TN	%
	Soil Total Carbon	TC	%
	Soil Total Phosphorus	TP	%
	Soil Calcium Carbonate Content	Soil CaCO_3_	g/kg
	Soil Inorganic Carbon	SIC	%
	Soil Organic Carbon	SOC	%
	Soil Organic Matter	SOM	g/kg

Individual structural traits of *S. breviflora* were measured directly. Leaf traits were assessed by scanning transparent plastic sheets containing leaves using a digital scanner set at 300 dpi. Scanned images were analyzed using ImageJ software to determine total leaf area and count the number of leaves measured. Before plant community surveys, plant height of *S. breviflora* within exclosure cages was measured. Subsequently, a 1m deep soil pit was excavated adjacent to each cage, and a controlled high-pressure water jet (water pressure ≤0.7 MPa) was used to wash soil from the cage area until individual *S. breviflora* plants were completely exposed for root collection. Root traits were analyzed using the WinRHIZO root scanning system. Total carbon and nitrogen content in *S. breviflora* roots, stems, and leaves were determined using a Vario MACRO CUBE elemental analyzer. Total phosphorus content in plant tissues was measured using a UV2300 UV-Vis spectrophotometer. Soil organic carbon (SOC) and total nitrogen (TN) were determined using the potassium dichromate-sulfuric acid external heating method and automated Kjeldahl digestion, respectively. Total phosphorus (TP) and available phosphorus (AP) were measured using the molybdenum-antimony colorimetric method following digestion and sodium bicarbonate extraction, respectively. Nitrate nitrogen (NO_3_^−^-N) and ammonium nitrogen (NH_4_^+^-N) were analyzed using UV spectrophotometry and indophenol blue colorimetry following KCl extraction, respectively. Soil inorganic carbon content was determined using a carbonate analyzer (Eijkeikamp) following NEN-ISO 10693 standards. Carbonate content was used to calculate soil inorganic carbon, which was then combined with total carbon data to determine organic carbon content and subsequently calculate soil organic matter content. Soil pH was measured using a Leici PHS-3C precision pH meter. Soil gravimetric water content (θg) and bulk density (BD) were determined using the oven-drying method.

Community-level metrics were derived from quadrat surveys. Species richness (SR) was calculated as the total number of plant species per quadrat. Community coverage (CC) was estimated visually as the total percentage of ground area covered by vegetation in each quadrat. Community height (CH) was measured as the average height of the vegetation canopy. Community aboveground biomass (C-AGB) was determined by clipping all aboveground plant material within the quadrat at ground level, sorting by species, drying at 65 °C to constant weight, and summing the dry weights of all species.

To assess the functional composition of the community, the community-weighted mean (CWM) of specific functional traits was calculated for each quadrat where trait data for constituent species were available. The CWM for a given trait was computed as:


$$CWM=\sum_{i=1}^{S}\left(p_{i}\times trait_{i}\right)$$

where *S* is the number of species in the quadrat, *p_i_*is the relative abundance (based on aboveground biomass) of species “*i*”, and *trait_i_* is the measured functional trait value for species “*i*”. Trait values for *S. breviflora* were obtained from our detailed direct measurements (Section 2.2). For all associated species, functional trait values were measured concurrently using the same protocols during the 2024 field campaign.

### 2.3 Data analysis

Data analysis was conducted using R version 4.2.1. Treatment differences were examined through one-way analysis of variance (ANOVA), while Pearson correlation analysis assessed relationships between indicators. Principal component analysis (PCA) was performed on the scaled (mean-centered and unit-variance) dataset of all measured community, plant, and soil variables to visualize the overall differentiation among stocking rate treatments and to identify major axes of ecosystem variation. Linear and nonlinear regression models were fitted to examine relationships between species richness and other key variables. Visualization was achieved through box plots, scatter plots, and PCA biplots. The significance level was set at *P* < 0.05, with highly significant differences defined as *P* < 0.01. The PCA was performed on a comprehensive dataset encompassing all measured community characteristics, functional traits of *S. breviflora*, and soil physicochemical properties (detailed in **Table 1**).

## 3 Results

### 3.1 Effects of different stocking rates on community characteristics and multi-organ traits of *S. breviflora* in desert steppe

**Figure 2:**
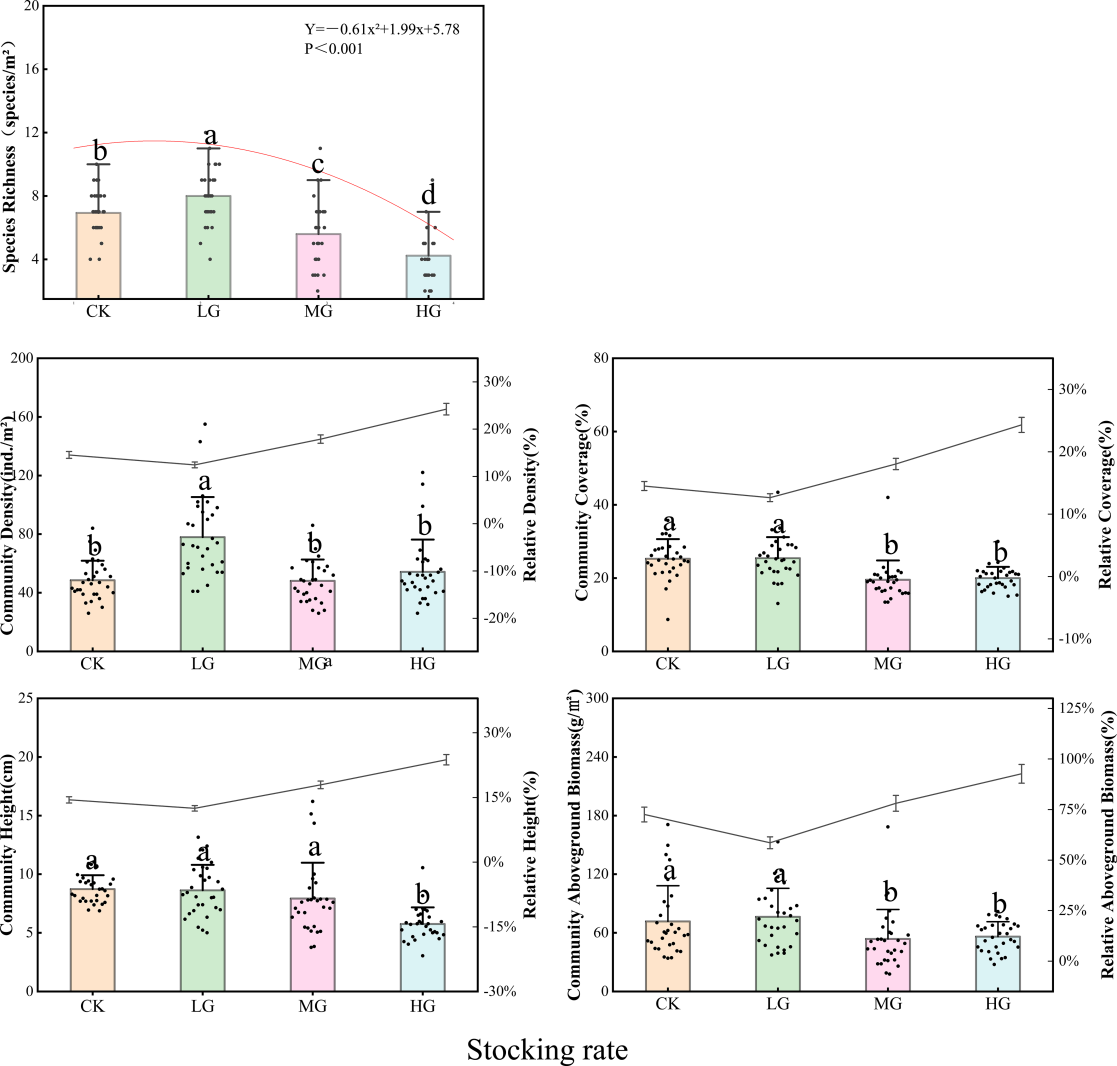
Response of community quantitative characteristics and relative dominance of *S. breviflora* to different stocking rates in desert steppe. Different lowercase letters above bars indicate significant differences among treatments at *P* < 0.05, the same below.

Results demonstrated that species richness, a key indicator of ecosystem structural stability, exhibited a unimodal response to stocking rate, with the highest richness under LG, followed by CK, and significant declines under MG and HG (**Figure 2**). Community density, another indicator of structural stability, was significantly higher in LG compared to CK, MG, and HG treatments. Community coverage and aboveground biomass (the latter characterizing ecosystem functional integrity) were significantly greater in CK and LG than in MG and HG. Community height, which reflects structural stability, was significantly lower in HG compared to CK, LG, and MG.

Relative density, relative coverage, relative height, and relative aboveground biomass of *S. breviflora* showed consistent response patterns, decreasing from CK to LG, then increasing progressively from LG through MG to HG. This indicates that relative dominance of *S. breviflora* increased gradually with increasing stocking rate.

In summary, stocking rate gradients significantly influenced both community characteristics and relative performance of the dominant species in *S. breviflora* desert steppe. Species richness, community density, coverage, aboveground biomass, and height exhibited distinct response patterns to increasing stocking rates. *S. breviflora* adapted to grazing disturbance by enhancing its relative dominance within the community (**Figure 2**).

**Figure 3:**
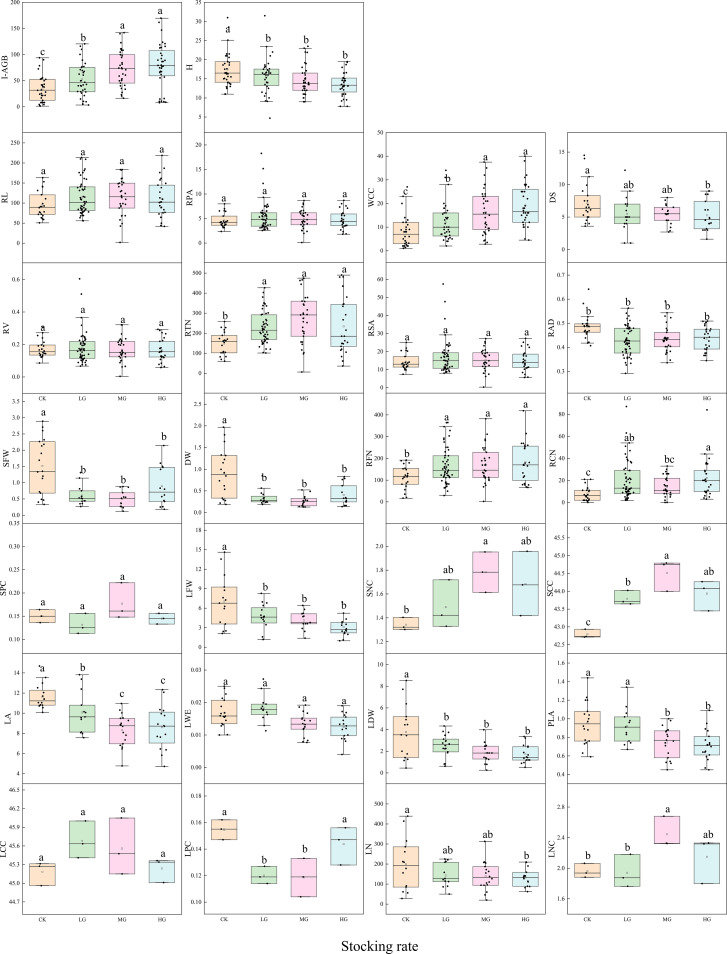
Box plots showing response differences in individual structure, root, stem, and leaf traits of *S. breviflora* under different stocking rates. The lowercase letters (a, b, ab, c) represent significant differences among different stocking rate treatments (CK, LG, MG, HG) at the P<0.05 level. Treatments with the same letter indicate no significant difference, while different letters indicate significant differences.

Analysis of variance revealed that different stocking rates exerted significant and systematic effects on individual structural traits, root characteristics, and stem and leaf functional traits of *S. breviflora*. Aboveground biomass, a core indicator of ecosystem functional integrity, increased significantly with treatment intensity (CK< LG< MG ≈ HG), whereas plant height, which reflects structural stability, showed the opposite trend (CK > LG ≈ MG ≈ HG). Crown width, an important component of structural traits, also expanded with increasing treatment intensity (CK< LG< MG ≈ HG), indicating that high-intensity treatments promoted lateral expansion rather than vertical growth, thereby adjusting the structural stability of the community. Stem base diameter was significantly lower in HG compared to CK, with no differences among other treatments, suggesting weak responses of stem thickness to treatment intensity. Among root morphological parameters, total root length, projected area, and surface area showed no significant differences among treatments. However, mean root diameter was significantly higher in CK than other treatments, while total root volume was significantly higher in HG than the other three groups. Root tip number and branching number were significantly higher in LG, MG, and HG compared to CK, with crossing number highest in HG, indicating that enhanced treatments primarily optimized resource acquisition through increased root branching complexity and volume rather than total length. The fresh and dry weights of stems and leaves were significantly greater in CK than in the other treatments, whereas total leaf area and individual leaf area showed the pattern CK ≈ LG > MG ≈ HG, demonstrating that high treatment intensity inhibited leaf biomass accumulation without significantly altering individual leaf quality. Nutrient element contents showed clear treatment dependence: nitrogen content in stems and leaves was highest in MG, followed by HG, with CK and LG lowest. Stem carbon content followed the pattern MG > LG ≈ HG > CK, while leaf carbon content showed LG ≈ MG > CK ≈ HG. Stem phosphorus content was highest in MG, followed by CK and HG, with LG lowest, whereas leaf phosphorus content showed CK > HG > LG ≈ MG, revealing differentiated carbon-nitrogen-phosphorus allocation strategies among organs. Overall, with increasing stocking rate, *S. breviflora* shifted its strategy from a ‘fast’-spectrum phenotype (higher aboveground biomass, lower root branching) toward a ‘slow’-spectrum phenotype (lower aboveground stature, higher root branching complexity and nutrient enrichment (**Figure 3**).

### 3.2 Integrated multivariate response and ecosystem strategy shift under long-term grazing

Principal component analysis (PCA) was conducted on a dataset integrating key variables from all three ecosystem components: community, dominant plant (*S. breviflora*) traits, and soil. This analysis aimed to capture the overarching multivariate response. The first two principal components (PCs) explained 21.4% (PC1) and 14.4% (PC2) of the total variance. The CK plots clustered distinctly in the positive region of PC1, whereas all grazing treatments distributed along the negative axis. LG occupied an intermediate position, while MG and HG treatments overlapped substantially in multivariate space. This convergence between MG and HG indicates a saturation in the overall ecosystem response beyond a specific stocking rate, visually defining a critical threshold.

Within the plant economic spectrum framework, PC1 loadings provided functional context for this multivariate shift (**Table 2**). PC1 represented a gradient from “fast” (acquisitive) to “slow” (conservative) resource-use strategies: the “fast” end is characterized by traits associated with high growth rates and community productivity, including indicators of community complexity and productivity (SR, CC, C-AGB), plant growth potential (LA), and soil resource availability (TN); while the “slow” end reflects traits geared towards persistence and stress tolerance under grazing, such as plant tissue defense and density (SNC/SCC, LNC), soil physical stress (BD), and a specific inhibitory nitrogen form (NO_3_^−^-N).

**Table 2:** Loadings of key variables on PC1 from the principal component analysis, representing a fast-to-slow plant economics spectrum.

Trait name	PC1 loading	Absolute loading	Strategy tendency	Contribution level
NO_3_^−^-N	-0.28946	0.28946	Slow strategy	High contribution
SNC	-0.27339	0.27339	Slow strategy	High contribution
SCC	-0.26446	0.26446	Slow strategy	High contribution
BD	-0.23448	0.23448	Slow strategy	High contribution
LNC	-0.21034	0.21034	Slow strategy	High contribution
SDW	0.20998	0.20998	Fast strategy	High contribution
LFW	0.20146	0.20146	Fast strategy	High contribution
TN	0.1943	0.1943	Fast strategy	High contribution
CC	0.19376	0.19376	Fast strategy	High contribution
SR	0.19208	0.19208	Fast strategy	High contribution
TC	0.18647	0.18647	Fast strategy	High contribution
H	-0.18505	0.18505	Slow strategy	Medium contribution
LA	0.17957	0.17957	Fast strategy	Medium contribution
PFW	0.17469	0.17469	Fast strategy	Medium contribution
PLA	0.17286	0.17286	Fast strategy	Medium contribution
SOC	0.16155	0.16155	Fast strategy	Medium contribution
I-AGB	-0.16093	0.16093	Slow strategy	Medium contribution
C	-0.15074	0.15074	Slow strategy	Medium contribution
CH	0.14556	0.14556	Fast strategy	Medium contribution
D	-0.14651	0.14651	Slow strategy	Medium contribution
SPC	-0.14386	0.14386	Slow strategy	Medium contribution
NH_4_^+^-N	0.1384	0.1384	Fast strategy	Medium contribution
RTN	-0.11479	0.11479	Slow strategy	Medium contribution
LPC	0.11381	0.11381	Fast strategy	Medium contribution
θg	-0.10078	0.10078	Slow strategy	Low contribution
Soil CaCO_3_	0.09778	0.09778	Fast strategy	Low contribution
SIC	0.09779	0.09779	Fast strategy	Low contribution
RFN	-0.07509	0.07509	Slow strategy	Low contribution
LCC	-0.07145	0.07145	Slow strategy	Low contribution
RCN	-0.06877	0.06877	Slow strategy	Low contribution
C-AGB	0.12317	0.12317	Fast strategy	Medium contribution
PH	0.06482	0.06482	Fast strategy	Low contribution
CD	0.0611	0.0611	Fast strategy	Low contribution
RAD	0.08351	0.08351	Fast strategy	Low contribution
RV	0.02336	0.02336	Fast strategy(weak)	Very low contribution
RL	-0.02789	0.02789	Slow strategy(weak)	Very low contribution
RPA	-0.00351	0.00351	NO obvious tendency	Very low contribution
RSA	-0.00351	0.00351	NO obvious tendency	Very low contribution
TP	-0.04087	0.04087	Slow strategy(weak)	Very low contribution
LWE	-0.03712	0.03712	Slow strategy(weak)	Very low contribution
SLA	-0.03284	0.03284	Slow strategy(weak)	Very low contribution
SFW	0.04521	0.04521	Fast strategy(weak)	Very low contribution

The systematic shift along PC1 demonstrated a grazing-driven ecosystem strategy transition. The ecosystem state progressed from the “fast”-strategy end (CK, positive scores) toward the “slow”-strategy end (HG, negative scores). The overlapping scores of MG and HG treatments signify that this strategic reorganization reaches saturation at MG, establishing it as a pivotal ecological threshold.

This integrative framework coherently explains key findings from specific univariate analyses. The decline in species richness (an archetypal “fast”-end trait) with increasing stocking rate (**Figure 2**) aligns with the overall shift toward a slower, less diverse state. Phenotypic adjustments in *S. breviflora*—reduced stature, decreased leaf biomass, and increased root branching and volume (Section 3.1)—represent functional reconfiguration toward a conservative, belowground-persistent strategy. Concurrently, grazing-induced soil modifications, including increased bulk density (a “slow”-end stressor) and altered nitrogen form ratios, create an abiotic filter that selects against “fast”-strategy species.

### 3.3 Trait-mediated linkages and the drivers of community responses

**Figure 4:**
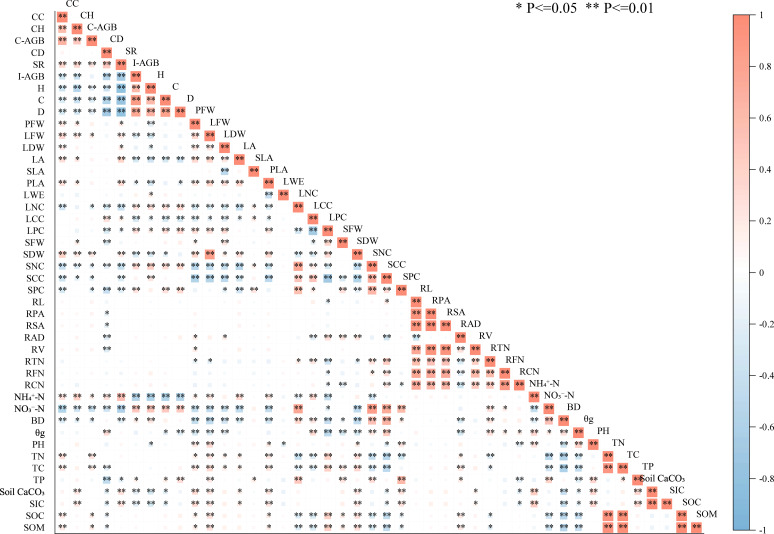
Heatmap of multi-indicator correlations in the “community-plant-soil” system of *S. breviflora* grassland under long-term grazing.

Correlation heatmap analysis revealed strong within- and cross-component associations (**Figure 4**). Significant positive correlations occurred within community characteristics (e.g., coverage, height, biomass, richness), among plant organ traits, and within soil properties. Critically, significant positive cross-component correlations linked community aboveground biomass (C-AGB) to plant aboveground traits (I-AGB) and soil nutrients (NH_4_^+^-N, TN). Root traits also correlated positively with I-AGB and soil TN. Soil TN correlated positively with community coverage (CC) and I-AGB but negatively with soil bulk density (BD).

The *S. breviflora* functional traits functioned as a key mediating nexus between soil nutrients (**Table 3**) and community characteristics. Mediation analysis confirmed that *S. breviflora* traits significantly mediated these relationships. The indirect effect via plant traits accounted for 63.2% of the total effect of soil NH_4_^+^-N on species richness (*P* < 0.01) and 58.7% of the total effect of soil TN on community coverage (*P* < 0.01). Coordinated adjustment of multi-organ traits (e.g., the synergistic effect between root branching and leaf nitrogen content) explained 47.3% of the variation in community-soil linkage strength (*P* < 0.01), with trait relative importance estimated at 45.6%.

**Table 3:** Soil physicochemical properties under different stocking rate treatments.

Soil properties	CK	LG	MG	HG
Soil Bulk Density	1.32 ± 0.05b	1.38 ± 0.04ab	1.41 ± 0.03a	1.39 ± 0.05ab
Soil Gravimetric Water Content	0.05 ± 0.01a	0.07 ± 0.02a	0.07 ± 0.01a	0.07 ± 0.01a
Soil Potential of Hydrogen	8.82 ± 0.12a	8.73 ± 0.14a	8.80 ± 0.09a	8.69 ± 0.09a
Soil Total Nitrogen	0.16 ± 0.02a	0.15 ± 0.02a	0.12 ± 0.02a	0.15 ± 0.01a
Soil Total Carbon	1.50 ± 0.21a	1.30 ± 0.25a	1.25 ± 0.17a	1.33 ± 0.10a
Soil Total Phosphorus	0.04 ± 0.002ab	0.04 ± 0.001b	0.04 ± 0.002a	0.04 ± 0.002b
Soil Calcium Carbonate Content	7.71 ± 3.36a	4.58 ± 3.46a	7.24 ± 5.09a	1.57 ± 1.03a
Soil Inorganic Carbon	0.09 ± 0.04a	0.05 ± 0.04a	0.09 ± 0.06a	0.02 ± 0.01a
Soil Organic Carbon	1.41 ± 0.17a	1.24 ± 0.24a	1.16 ± 0.23a	1.31 ± 0.10a
Soil Organic Matter	24.32 ± 2.98a	21.41 ± 4.19a	20.00 ± 3.89a	22.65 ± 1.64a
Soil Ammonium Nitrogen	0.70 ± 0.001b	0.78 ± 0.01a	0.70 ± 0.01b	0.50 ± 0.001c
Soil Nitrate Nitrogen	0.92 ± 0.001c	0.79 ± 0.003d	1.69 ± 0.001a	1.45 ± 0.001b

Different letters indicate significant differences at P< 0.05.

**Figure 5:**
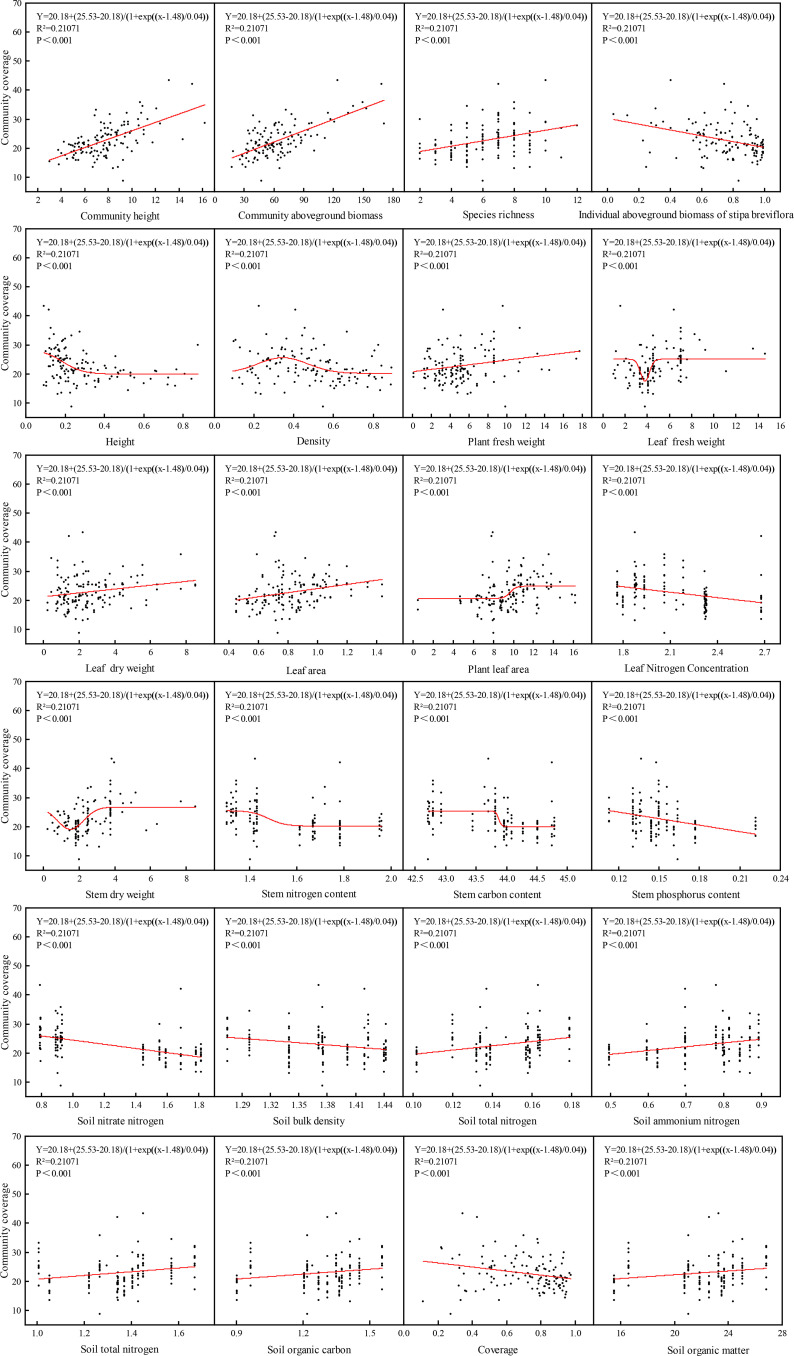
Regression analysis of species richness with plant and soil indicators in *S. breviflora* desert steppe.

Species richness (SR) responded to multiple biotic and abiotic drivers (**Figure 5**). SR exhibited a significant positive correlation with soil ammonium nitrogen (NH_4_^+^-N) but was strongly inhibited by soil bulk density (BD) and nitrate nitrogen (NO_3_^−^-N). SR correlated negatively with indicators of *S. breviflora* dominance, including individual aboveground biomass (I-AGB), plant height (H), stem phosphorus content (SPC), and community coverage (CC). Positive effects of leaf fresh weight (LFW) and individual leaf area (LA) on SR showed saturation thresholds at higher values.

**Figure 6:**
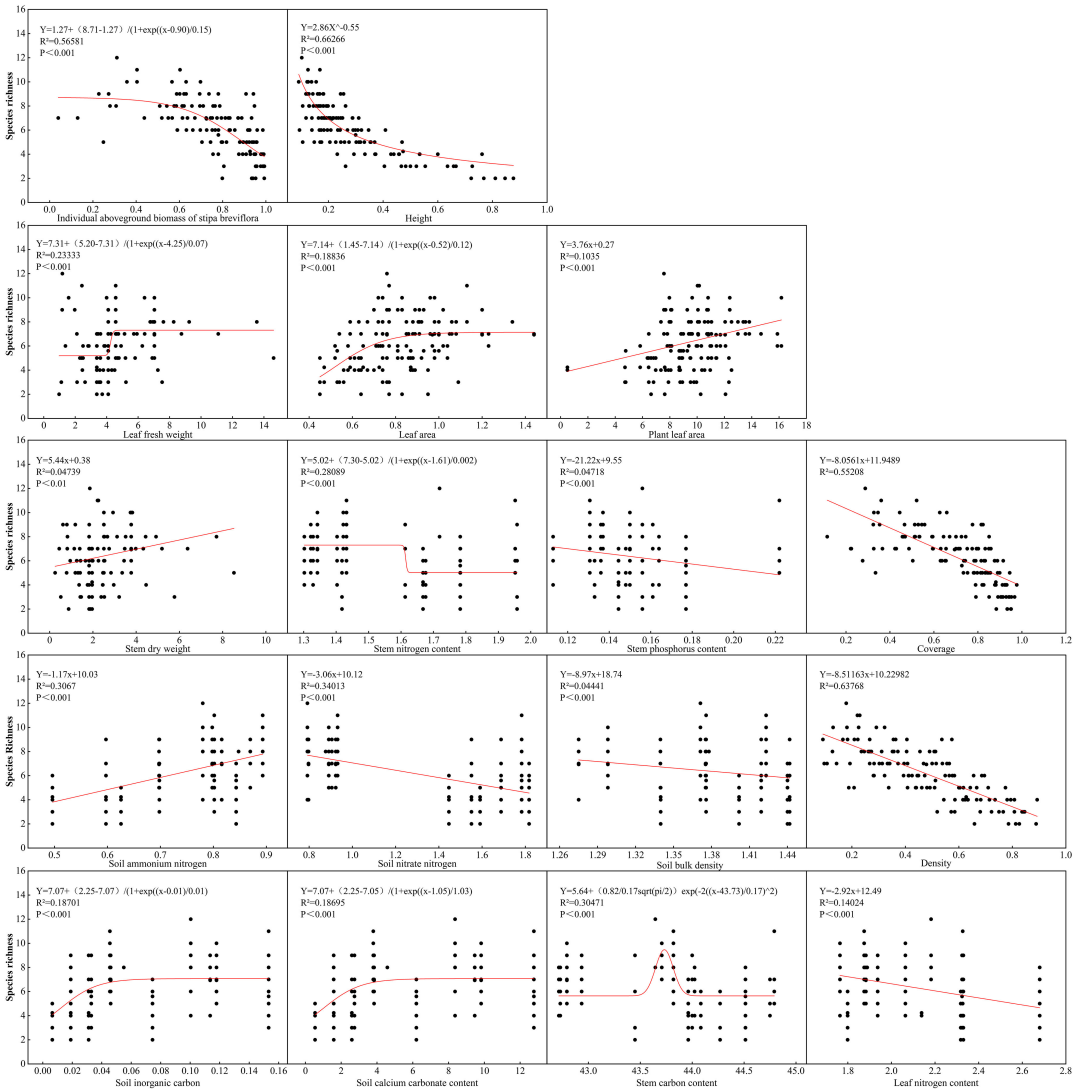
Regression analysis of community coverage with multiple factors in *S. breviflora* desert steppe.

Community coverage (CC) was regulated by multiple factors with distinct patterns (**Figure 6**). CC increased linearly with community height (CH), aboveground biomass (C-AGB), and soil total nitrogen (TN). It was negatively affected by soil nitrate (NO_3_^−^-N) and by *S. breviflora* individual biomass (I-AGB) and tissue nitrogen content (LNC, SNC). Relationships with stem dry weight (SDW), stem carbon content (SCC), and plant leaf area (PLA) were positive but saturating. Correlations with SR, BD, SOC, SOM, NH_4_^+^-N, LA, or PFW were negligible.

**Figure 7:**
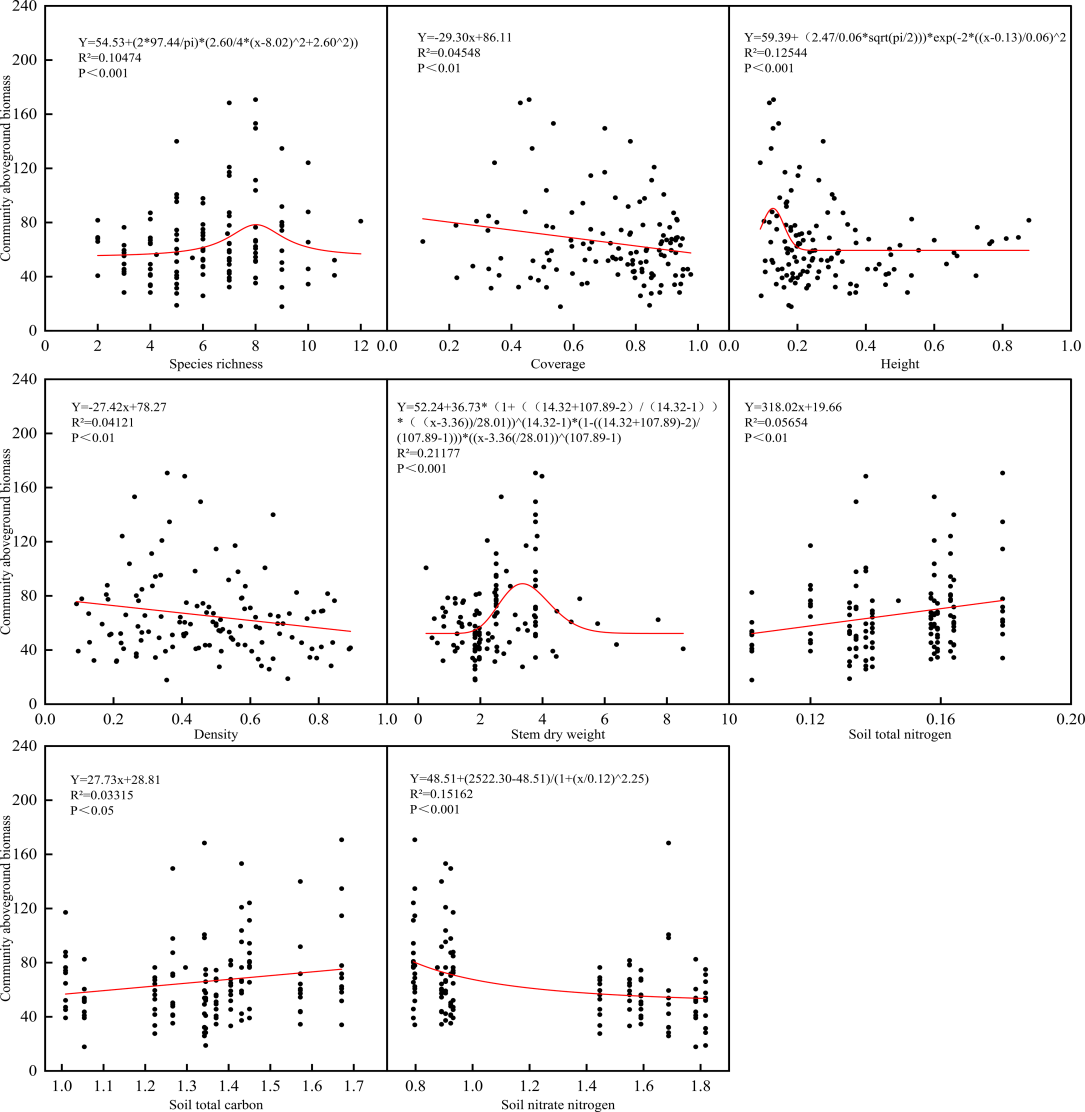
Regression analysis of community aboveground biomass with community, plant and soil indicators in *S. breviflora* desert steppe.

Community aboveground biomass (C-AGB) displayed non-linear relationships central to productivity trade-offs (**Figure 7**). C-AGB exhibited unimodal relationships with both species richness (SR) and plant height (H), peaking at intermediate values. It showed significant negative correlations with community density (D) and coverage (CC). Stem dry weight (SDW) displayed a non-linear “increase then stabilization” relationship with C-AGB. Among soil factors, C-AGB showed weak positive correlations with TN and TC, and a distinct non-linear “decrease then stabilization” response to soil NO_3_^−^-N, remaining consistently low when NO_3_^−^-N exceeded 1.0 mg/kg.

**Figure 8:**
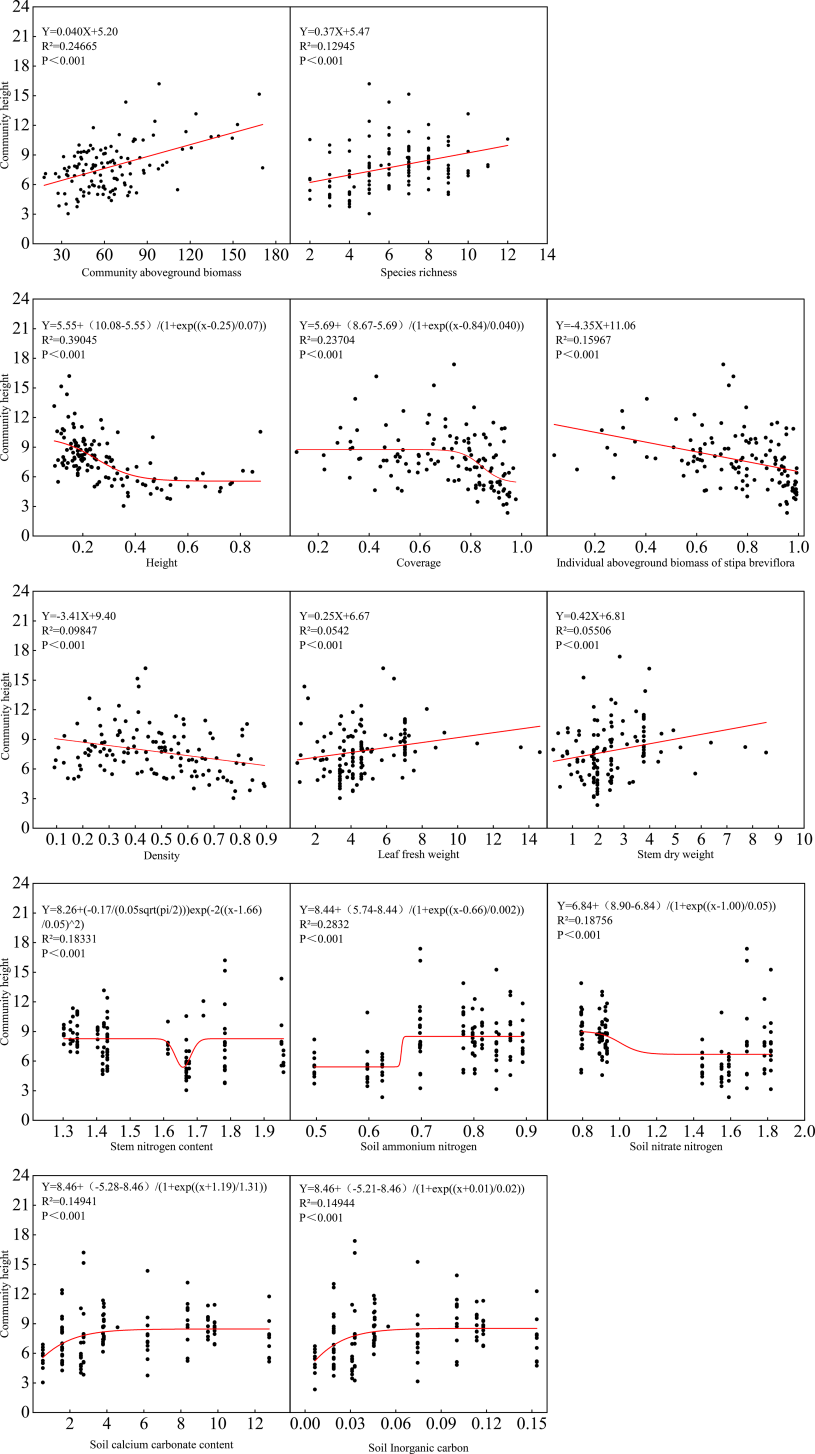
Regression analysis of community height with community, plant and soil indicators in *S. breviflora* desert steppe.

Community height (CH) was shaped by plant size and competitive interactions (**Figure 8**). CH correlated significantly positively with C-AGB and showed a non-linear “increase then stabilization” relationship with individual plant height. It exhibited a unimodal response to community coverage (CC). CH correlated negatively with *S. breviflora* individual aboveground biomass (I-AGB) and community density (D), but positively with stem dry weight (SDW). Soil ammonium (NH_4_^+^-N) and nitrate (NO_3_^−^-N) nitrogen, as well as stem nitrogen content (SNC), showed non-linear “decrease then stabilization” inhibitory effects on CH. Soil calcium carbonate and inorganic carbon exhibited “increase then stabilization” effects.

These regression analyses confirm that the integrated strategic shift is mechanistically driven by: (i) abiotic soil changes (increased BD, elevated NO_3_^−^-N/NH_4_^+^-N ratio) creating a stress filter; (ii) coordinated trait adjustments in *S. breviflora* toward a conservative phenotype; and (iii) intensified biotic competition leading to community simplification. The convergence of response trajectories for multiple indicators under MG and HG treatments reinforces the existence of the critical stocking rate threshold identified in the multivariate analysis.

## 4 Discussion

### 4.1 Grazing effects on community structure and diversity

As described in Methods, our data represent a snapshot after 20 years of consistent treatments, which we interpret as reflecting long-term grazing equilibria. This study definitively shows that *S. breviflora* desert steppe exhibits core patterns of continuous species richness decline accompanied by synchronous increases in relative dominance of the dominant species under gradient stocking rates. This phenomenon is rooted in the unique resource competition-phenotypic plasticity coupling mechanism characteristic of arid ecosystems (Li et al., 2019).

Desert steppes experience scarce annual precipitation and poor soil conditions. Although light grazing temporarily increased community density (possibly related to moderate disturbance releasing spatial resources), it disrupted the fundamental resource balance supporting species coexistence (Wu J-X, et al., 2022). As stocking rates further increased, selective grazing by livestock and trampling effects synergistically reinforced the competitive advantage of *S. breviflora*. This species efficiently captured limited water and nitrogen resources through phenotypic adjustments, while simultaneously suppressing establishment and growth of other species through dense canopy shading, ultimately forming the stepwise decline pattern of species richness following LG > CK > MG > HG (Lv et al., 2020). Although temporal dynamics of species richness could not be directly analyzed, the proportion of perennial grasses (the dominant functional group) decreased from CK to HG, while the proportion of annual forbs increased from CK to HG, indicating a functional group replacement process driven by long-term grazing, which indirectly reflects the dynamic degradation of community diversity.

This response pattern contrasts sharply with the classic intermediate disturbance theory promoting diversity in mesic grasslands, primarily because desert steppe ecosystems have extremely low resilience thresholds, where any herbivore pressure easily exceeds the system’s compensatory capacity, preventing associated species from completing their life cycles within the brief growing season (Meyer et al., 2021; Zhang et al., 2026). The complex associations between species richness and multiple ecological factors clearly reveal the dual regulation logic of resource competition-environmental filtering underlying biodiversity maintenance in arid regions (Hou et al., 2023). Individual aboveground biomass of dominant species and community coverage showed the strongest explanatory power for species richness—high-biomass individuals monopolized light, water, and nutrient resources through canopy shading and root expansion, progressively excluding associated species and directly constraining community species coexistence.

Community coverage, as a key component of community structure, is governed by a three-tier mechanism that integrates resource supply, competition intensity and adaptive strategy. A moderate positive relationship between soil total nitrogen and community coverage confirms N as the primary limiting resource for canopy development across arid regions, a pattern repeatedly reported for the loess hilly grasslands. In contrast, soil nitrate nitrogen exerts a consistent negative effect; in alkaline soils the combination of ionic toxicity and osmotic stress reduces coverage, a outcome that diverges sharply from the facilitative role of nitrate in mesic steppes where water is non-limiting. The weak negative correlation between individual above-ground biomass and tissue nitrogen content of *S. breviflora* and community coverage indicates that large individuals, by developing deep roots and expanded crowns, monopolize limited water and nitrogen and thereby suppress subordinate species, a mechanism that explains the low-cover, mono-dominant stands observed along grazing gradients on the Mongolian Plateau but contradicts the positive dominance–coverage relationship typical of high-productivity grasslands.

Community height, another critical structural attribute, is primarily driven by individual development processes of dominant species rather than synergistic effects of species coexistence (Jia et al., 2019). The significant positive correlation between community height and community aboveground biomass reflects that enhanced productivity is accompanied by vertical structural development (Chondol et al., 2025). However, individual aboveground biomass of *S. breviflora* and community density both showed significant negative correlations with community height, reflecting that intensified resource competition constrains individual elongation. This study quantitatively elucidated the nonlinear threshold relationship between dominant species expansion and diversity decline under gradient stocking rates, providing quantitative basis for determining critical stocking rates in arid grassland management.

### 4.2 Coordinated multi-organ trait responses of *Stipa breviflora*

Across the gradient stocking rates, *S. breviflora* adopted an aboveground-contraction–underground-reinforcement strategy, a conservative life-history mechanism adapted to herbivore pressure in arid desert steppes. As stocking rate increased, *S. breviflora* exhibited a suite of coordinated trait shifts, including reduced aboveground stature and leaf biomass alongside increased root branching complexity and volume (Section 3.1). This coordinated adjustment of aboveground traits—reduced stature to avoid herbivory and expanded crown width to enhance resource capture—reflects the species’ adaptive trade-off under grazing disturbance.

Root system traits showed equally distinctive response patterns: root branching complexity indicators including root tip number and branching number were significantly elevated by 30-50% in grazing treatments compared to CK. Among root morphological parameters, total root volume was significantly higher in HG than the other three groups, with crossing number highest in HG, indicating that enhanced grazing treatments primarily optimized resource acquisition through increased root branching complexity and volume rather than total length. The core of this morphological plasticity adjustment involves reallocating limited resources from aboveground parts vulnerable to grazing toward better-protected underground organs and horizontal expansion structures, thereby maintaining water and nutrient acquisition capacity (Siebenkäs and Roscher, 2016).

Nutrient element contents in different organs showed clear treatment dependence, reflecting coordinated nutrient allocation strategies. Nitrogen content in stems and leaves was highest in MG, followed by HG, with CK and LG lowest. Stem carbon content followed the pattern MG > LG ≈ HG > CK, while leaf carbon content showed LG ≈ MG > CK ≈ HG. Stem phosphorus content was highest in MG, followed by CK and HG, with LG lowest, whereas leaf phosphorus content showed CK > HG > LG ≈ MG. These differentiated carbon-nitrogen-phosphorus allocation strategies among organs highlight the species’ ability to optimize resource use efficiency under varying grazing pressures.

Under light to moderate grazing disturbance, *S. breviflora* prioritized optimization of nutrient use efficiency, with stem and leaf nitrogen and carbon contents reaching peaks of 1.82% and 45.3% respectively under moderate treatment. Under heavy grazing conditions, although biomass decreased, root volume and crossing number significantly increased by 42.6% and 38.9% compared to CK, indicating the existence of stocking rate-dependent physiological thresholds where extreme pressure ensures survival through enhanced root spatial occupation (Ma et al., 2023). This conservative defense strategy is better adapted to the extremely poor resource environment of desert steppes compared to rapid regeneration strategies of mesic grassland plants, as it more efficiently enhances plant fitness (Zheng et al., 2024). Overall, with increasing treatment intensity, *S. breviflora* shifted from a conservative strategy characterized by high biomass and low branching to an acquisitive strategy featuring low aboveground biomass, high root branching, and nutrient enrichment. This study comprehensively analyzed the complete spectrum of multi-organ trait responses to grazing at the individual level, confirming that dominant species can maintain ecosystem stability through functional trait plasticity, providing physiological ecological support for predicting community succession direction driven by grazing.

### 4.3 Grazing-induced changes in soil nitrogen dynamics

Soil nitrogen dynamics, as a core component of the desert steppe ecosystem, showed significant responses to long-term grazing disturbance. Soil total nitrogen (TN) showed significant positive correlations with total carbon (TC) and soil organic matter (SOM), while soil bulk density (BD) showed significant negative correlations with soil gravimetric water content (θg) and total nitrogen (TN), highlighting that soil physical structure plays a key role in regulating nitrogen availability. The formation of this cascade regulation relationship stems from the habitat characteristics of poor soil and intense evaporation in desert steppes, which make nitrogen mineralization and retention highly dependent on soil microstructural stability (Han et al., 2017).

Different forms of inorganic nitrogen exhibited contrasting responses to grazing gradients. Soil ammonium nitrogen (NH_4_^+^-N) demonstrated significant positive effects on species richness, whereas soil nitrate nitrogen (NO_3_^−^-N) exhibited significant inhibitory effects. This differentiation reflects the special regulatory functions of nitrogen forms under alkaline soil backgrounds: ammonium nitrogen is more readily absorbed by various plants, providing a nutrient basis for species coexistence, while nitrate nitrogen is prone to leaching or accumulation to stress concentrations, further strengthening the competitive exclusion effect of dominant species (Wu W, et al., 2022). Specifically, soil ammonium nitrogen showed a significant positive association with species richness, while soil nitrate nitrogen exerted inhibitory effects, which was particularly evident in the “decrease then stabilization” relationship between community aboveground biomass and soil nitrate nitrogen-biomass remained consistently low when nitrate nitrogen exceeded 1.0 mg/kg, stemming from ion toxicity and osmotic stress induced by nitrate nitrogen in alkaline soils.

Stocking rate also altered soil nitrogen availability through trampling effects: soil bulk density increased with stocking rate, which not only impedes seed germination and seedling establishment but also reduces water infiltration through decreased porosity, forming a rigid barrier to nitrogen cycling and species diversity loss (Hong et al., 2010). The strong negative effect of soil bulk density on species richness further confirms that soil physical structure, through its regulation of nitrogen availability, constitutes a core factor constraining ecosystem function in the arid context of loess hilly regions (Bui et al., 2024). These findings highlight that grazing-induced changes in soil nitrogen dynamics—including both total nitrogen content and nitrogen form transformation—play a pivotal role in mediating ecosystem structural and functional responses to long-term grazing disturbance.

### 4.4 Trait-mediated linkages among community, plant, and soil components

**Figure 9:**
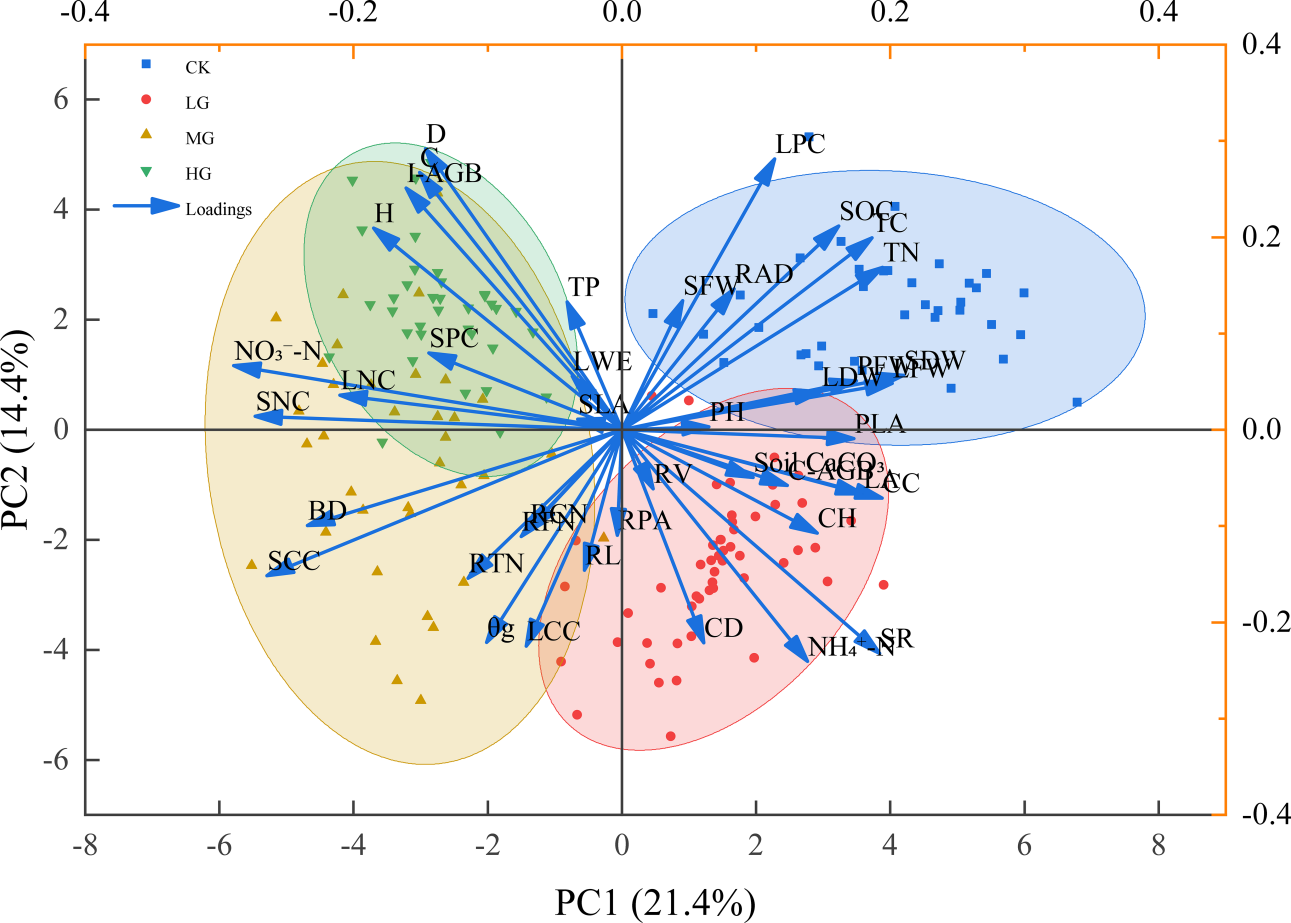
Principal component analysis (PCA) biplot of multi-indicator responses under different stocking rates in *S. breviflora* desert steppe.

Collectively, our findings provide direct answers to the three scientific questions outlined in the introduction. (1) Under long-term gradient stocking rates, the linkage relationships among community diversity, *S. breviflora* multi-organ traits, and soil nitrogen cycling are characterized by tightly coupled, non-linear associations, with the overall multi-component system shifting coordinately along a ‘fast’ to ‘slow’ plant economics spectrum (PCA, **Figure 9**; **Table 2**). (2) The stocking rate threshold, identified at the moderate grazing (MG) level, regulates the direction and intensity of these linkages by triggering a saturated ecosystem response; beyond this threshold (i.e., under MG and HG), further increases in grazing pressure do not alter the established ‘slow’-strategy state dominated by *S. breviflora*, thereby decoupling further directional change in linkage patterns. (3) The coordinated multi-organ trait adjustments of *S. breviflora* —specifically the shift towards reduced aboveground stature, increased root branching, and altered tissue nutrient concentrations—are confirmed as the key functional nexus connecting soil changes to community outcomes. This is evidenced by mediation analysis showing that *S. breviflora* traits mediated a substantial proportion (e.g., 63.2%) of the effect of soil nitrogen on species richness.

Correlation heatmaps and principal component analysis (PCA) collectively demonstrated the tightly integrated nature of the *S. breviflora* desert steppe ecosystem, where trait-mediated pathways of *S. breviflora* serve as the central functional nexus connecting community structure, plant individual traits, and soil nutrient dynamics (Lv et al., 2021; Zhang et al., 2018). Rather than operating independently, these three components form functionally coupled networks through coordinated multi-organ trait adjustments of the dominant species *S. breviflora*, reflecting the synergistic adaptation strategy dominated by resource limitation in arid environments.

At the cross-component level, significant positive correlations were identified between community aboveground biomass (C-AGB) and key plant aboveground traits (e.g., individual aboveground biomass, leaf fresh weight) as well as critical soil nutrients (e.g., ammonium nitrogen, total nitrogen). Root morphological traits of *S. breviflora*, including root length and root projected area, also exhibited strong positive correlations with both individual aboveground biomass and soil nutrient availability, confirming the tightly coupled relationships among the three components. Notably, soil total nitrogen showed robust positive correlations with both community coverage and individual plant biomass, highlighting that soil nitrogen availability—regulated by soil physical structure—acts as a core driver of the trait-mediated linkage network.

PCA further quantitatively characterized the differentiation of trait-mediated linkage patterns across different stocking rates (Wang et al., 2024). Control (CK) samples clustered in the positive direction of PC1, reflecting the synergistic characteristics of high community coverage, high species diversity, and favorable soil health status. In contrast, light grazing (LG), moderate grazing (MG), and heavy grazing (HG) samples were sequentially distributed along the negative PC1 axis, with a progressive shift toward the negative end as stocking rate increased. The substantial overlap between MG and HG samples indicated that ecosystem response patterns tend to converge under moderate-to-heavy grazing, a phenomenon attributed to the stocking rate threshold effect. Beyond this threshold, *S. breviflora* responds to sustained grazing pressure by enhancing root expansion and underground resource monopolization, triggering an ecological simplification process centered on competitive exclusion. This is manifested in the synergistic occurrence of species diversity loss and ecosystem functional degradation, empirically verifying that long-term grazing-induced stocking rate thresholds reshape trait-mediated linkage patterns.

The complexity of these trait-mediated linkages was further revealed by nonlinear response relationships between community aboveground biomass and multiple biotic and abiotic factors. Unimodal relationships were observed between C-AGB and both species richness and plant height, indicating that moderate species diversity and individual plant height optimize resource use efficiency through niche complementarity while avoiding productivity losses from excessive competition under high diversity. Additionally, stem dry weight of *S. breviflora* showed an “increase-then-stabilization” nonlinear pattern with C-AGB, reflecting the trade-off in plant resource allocation—moderate investment in structural biomass supports the expansion of photosynthetic organs and enhances carbon assimilation potential, whereas excessive lignification consumes substantial carbon resources and reduces overall carbon assimilation efficiency (Hu et al., 2026). These findings update the traditional understanding of diversity-productivity relationships, confirming that trait-mediated linkages among community, plant, and soil components are jointly regulated by competitive intensity and resource availability, with stocking rate thresholds modifying the strength and direction of these linkages.

### 4.5 Limitations and future perspectives

A limitation of our study is the reliance on data from a single growing season. While we argue it represents a long-term equilibrium state, future work incorporating interannual replication would strengthen the mechanistic inferences and help disentangle treatment effects from annual variability.

This study also recognizes limitations in exploring trait-mediated linkages, including the lack of differentiation in responses among different plant functional groups, insufficient integration of belowground interaction processes, and inadequate analysis of early recovery community succession dynamics. Future research should combine functional trait spectrum analysis, microbiome research methods, and long-term positioning observations to decipher the molecular regulatory mechanisms underlying the phenotypic plasticity of *S. breviflora*. Additionally, verifying the cascade pathway of “soil physical structure-microbial activation-plant adaptive strategy” and tracking the dynamic evolution of trait-mediated linkages across different recovery stages will be critical. Microcosm control experiments and microtopography modification studies could further investigate the feedback effects of root architecture changes on soil structure and the causal role of soil structure improvement in linkage reconstruction, ultimately breaking through the constraints of monodominant communities on biodiversity recovery and optimizing precision management and sustainable restoration strategies for arid grassland ecosystems (Li et al., 2025).

## 5 Conclusions

This study shows that long-term gradient stocking rates significantly drive synergistic responses across the “community-plant-soil” multi-component system in *S. breviflora* desert steppe. Under long-term gradient stocking rates, species richness showed a stepwise decline with increasing stocking rate, representing the steady-state response of community diversity after 20 years of continuous grazing disturbance. Due to the limitation of single-year data, the temporal dynamics of species diversity could not be fully elucidated, and this will be addressed in future long-term monitoring studies. *S. breviflora* enhanced its relative dominance through phenotypic plasticity, including reduced aboveground stature, decreased leaf biomass, and increased root branching number and root volume. Nonlinear associations were observed among the multi-component system, with moderate grazing serving as the critical threshold where system responses converged under MG and HG treatments. Soil ammonium nitrogen demonstrated significant positive effects on species richness, whereas soil nitrate nitrogen and bulk density exhibited significant inhibitory effects.

## Data Availability

The raw data supporting the conclusions of this article will be made available by the authors, without undue reservation.

## References

[B1] Bui T. T. X. (2024). Plant growth and adaptive responses to Cd stress under effects of nitrogen nutrient: A review. Res. J. Biotechnol. 19, 112–121. doi: 10.25303/1908rjbt1120121

[B2] Chai Y. Cao Y. Yue M. Tian T. Yin Q. Dang H. . (2019). Soil abiotic properties and plant functional traits mediate associations between soil microbial and plant communities during a secondary forest succession on the Loess Plateau. Front. Microbiol. 10, 895. doi: 10.3389/fmicb.2019.00895, PMID: 31105679 PMC6499021

[B3] Chondol T. Klimeš A. Hiiesalu I. Altman J. Čapková K. Jandová V. . (2025). Contrasting habitat associations and ecophysiological adaptations drive interspecific growth differences among Himalayan high-mountain plants. Ann. Bot. 135, 1093–1106. doi: 10.1093/aob/mcaf014, PMID: 39868547 PMC12259540

[B4] Han M. Pan Z. Jin Y. Qin J. Li J. Wang Z. . (2017). Response of soil nitrogen mineralization to different stocking rates on the *S. breviflora* desert steppe. Acta Prataculturae Sin. 26, 27–35. doi:10.11686/cyxb2017118

[B5] Hong L. I. Su-fang F. A. N. Guang-can Z. Shu-yong Z. Ze-fu Z. (2010). Characteristics of Soil Water-Holding and Soil Porosity under Different Tree Species after Conversion of Cropland to Forest in the Loss Hilly Region. Bull. Soil Water Conserv. 30, 75–81. doi: 10.13961/j.cnki.stbctb.2010.01.017

[B6] Hou G. Zong N. Shi P. (2023). Shifts in dominant species modulate nitrogen effects on community temporal stability along a degradation gradient in Tibetan alpine grasslands. Ecol. Indicators. 154, 110650. doi: 10.1016/j.ecolind.2023.110650, PMID: 41737640

[B7] Hu M. Shi H. He R. Wang N. Han Y. Dang H. . (2026). Tree biomass allocation is governed by allometry but modulated by optimization. For. Ecosystems. 15, 100405. doi: 10.1016/j.fecs.2025.100405, PMID: 41737640

[B8] Jia H. Chen Y. Wang X. Li P. Yuan Z. Ye Y. (2019). The relationships among topographically-driven habitats, dominant species and vertical layers in temperate forest in China. Russian J. Ecology. 50, 172–186. doi: 10.1134/S1067413619020061, PMID: 41411595

[B9] Ju X. Wang B. Wu L. Zhang X. Wu Q. Han G. (2024). Grazing decreases net ecosystem carbon exchange by decreasing shrub and semi-shrub biomass in a desert steppe. Ecol. Evolution. 14, e11528. doi: 10.1002/ece3.11528, PMID: 38932943 PMC11199334

[B10] Li J. Xu C. Zhu J. Zhao K. (2019). Resource trade-off in Cotinus coggygria seedlings under drought conditions – phenotypic plasticity of leaf and fine root functional traits. Int. J. Agric. Biol. 22, 683–692. doi: 10.17957/IJAB/15.1116

[B11] Li J. Han G. Kang S. Zhang X. Li C. (2022). Responses of tillering S. breviflora traits to a Long-term grazing gradient. Acta Societatis Botanicorum Poloniae. 91, 913. doi: 10.5586/asbp.913

[B12] Li Q. Wang R. Wang H. Jiang H. Yuan Y. Fu G. . (2024). Characteristics of dust emission over desert steppe. Geoderma 448, 116958. doi: 10.1016/j.geoderma.2024.116958, PMID: 41737640

[B13] Li R. Wang H. Yan Y. Han C. Zhao Y. Liu Y. . (2025). Effects on soil structures by root growth in the Loess Plateau, China. CATENA 261, 109536. doi: 10.1016/j.catena.2025.109536, PMID: 41737640

[B14] Liu Y. Pan Q. Liu H. Bai Y. Simmons M. Dittert K. . (2011). Plant responses following grazing removal at different stocking rates in an Inner Mongolia grassland ecosystem. Plant Soil. 340, 199–213. doi: 10.1007/s11104-010-0458-3, PMID: 41737715

[B15] Lv S. Huang J. Liu H. Ma S. (2024). Grazing effects on species diversity across different scales are related to grassland types. BMC Plant Biol. 24, 1103. doi: 10.1186/s12870-024-05812-z, PMID: 39567892 PMC11577882

[B16] Lv G. Wang Z. Guo N. Xu X. Liu P. Wang C. (2021). Status of S. breviflora as the constructive species will be lost under climate change in the temperate desert steppe in the future. Ecol. Indicators. 126, 107715. doi: 10.1016/j.ecolind.2021.107715, PMID: 41737640

[B17] Lv S. Yan B. Wang Z. Wang Z. Song X. Zhao M. . (2020). Dominant species’ dominant role and spatial stability are enhanced with increasing stocking rate. Sci. Total Environment. 730, 138900. doi: 10.1016/j.scitotenv.2020.138900, PMID: 32388367

[B18] Ma W. Ding K. Bai S. Wang C. Droma T. (2023). Response of bacterial communities to shrub encroachment and forage planting in alpine grassland of the Qinghai-Tibetan plateau. Ecol. Engineering. 186, 106837. doi: 10.1016/j.ecoleng.2022.106837, PMID: 41737640

[B19] Meyer P. Schmidt M. Feldmann E. Willig J. Larkin R. (2021). Long-term development of species richness in a central European beech (Fagus sylvatica) forest affected by windthrow—Support for the intermediate disturbance hypothesis? Ecol. Evolution. 11, 12801–12815. doi: 10.1002/ece3.8028, PMID: 34594540 PMC8462171

[B20] Siebenkäs A. Roscher C. (2016). Functional composition rather than species richness determines root characteristics of experimental grasslands grown at different light and nutrient availability. Plant Soil. 404, 399–412. doi: 10.1007/s11104-016-2853-x, PMID: 41737715

[B21] Valani G. P. Martíni A. F. MaChado C. B. Duarte D. A. de Brito M. F. P. Pezzopane J. R. M. . (2025). Soil porosity in integrated and non-integrated grazing systems in a Brazilian Ferralsol assessed by 3D X-ray computed tomography. Comput. Electron. Agriculture. 237, 110557. doi: 10.1016/j.compag.2025.110557, PMID: 41737640

[B22] Wang M. Ma C. (1994). Study on estimating grassland carrying capacity by two methods. Chin. J. Grassland 5, 19–22. doi: CNKI:SUN:ZGCD.0.1994-05-004

[B23] Wang X. Song N. Yang X. Wang L. Chen L. (2018). Grazing exclusion-induced shifts, the relative importance of environmental filtering, biotic interactions and dispersal limitation in shaping desert steppe communities, northern China. J. Arid Land. 10, 402–415. doi: 10.1007/s40333-018-0411-5, PMID: 41737715

[B24] Wang Y. Wang Z. Wu L. Li H. Li J. Zhu A. . (2024). Effects of grazing and climate change on aboveground standing biomass and sheep live weight changes in the desert steppe in Inner Mongolia, China. Agric. Systems. 217, 103916. doi: 10.1016/j.agsy.2024.103916, PMID: 41737640

[B25] Wei Z. Han G. Yan J. Lv X. (2000). Responses of plant community characteristics of Stipa breviflora desert steppe to different stocking rates. Chin. J. Grassland 6, 2–26. doi: 10.3321/j.issn:1673-5021.2000.06.001, PMID: 30704229

[B26] Wu J.-X. Li S.-Y. Han G.-D. (2022). Linkage between soil stoichiometric imbalance and microbial carbon use efficiency in desert steppe under different grazing intensities. J. Appl. Ecol. 35, 2108–2118. doi: 10.13287/j.1001-9332.202408.003, PMID: 39419796

[B27] Wu W. Wang X. Ren Z. Zhou X. Du G. (2022). N-induced species loss dampened by clipping mainly through suppressing dominant species in an alpine meadow. Front. Plant Science. 13, 815011. doi: 10.3389/fpls.2022.815011, PMID: 35392523 PMC8980528

[B28] Zhang C. Dong Q. Chu H. Shi J. Li S. Wang Y. . (2018). Grassland community composition response to grazing intensity under different grazing regimes. Rangeland Ecol. Management. 71, 196–204. doi: 10.1016/j.rama.2017.09.007, PMID: 41737640

[B29] Zhang F. Xu L. Li S. Zheng J. Yang L. Zhang B. . (2026). Legacy of heavy grazing triggers faster natural recovery in degraded temperate steppe. Agriculture Ecosyst. Environment. 397, 110067. doi: 10.1016/j.agee.2025.110067, PMID: 41737640

[B30] Zheng J. Wang Q. Yuan S. Zhang B. Zhang F. Li S. . (2024). Soil deterioration due to long-term grazing of desert-steppe promotes stress-tolerant ecological strategies in plants. Sci. Total Environment. 907, 168131. doi: 10.1016/j.scitotenv.2023.168131, PMID: 39491197

